# 

**Published:** 1892-04

**Authors:** 


### TABLE OF CONTENTS.

ORIGINAL COMMUNICATIONS. PAGE

Pyorrhœa Alveolaris due largely to Systemic Predisposition. By C. N. Peirce, D.D.S. . . . . . 241

Pulp-Stones: Their Significance, Methods of Diagnosis, and Necessary Treatment. By Dr. Rodriguez Ottolengui, New York. . . . . . 248

Pulpless Teeth; Abscess; Treatment, especially Surgical Treatment. By J. M. Porter, D.D.S., New York . . . . . . 255

Toothache: Its Diagnosis and Treatment. By Dr. A. V. Elliott, Florence, Italy . . . . . . 263

Soft Gold-Foil as a Filling-Material. By Dr. C. H. Gerrish, Exeter, N. H. 268

A New Preparation of Gutta-Percha. By William H. Rollins, Boston 272

REPORTS OF SOCIETY MEETINGS.

New York Odontological Society . . . . . . 273

American Dental Association . . . . . . 286

Odontological Society of Pennsylvania . . . . . . 292

Central Dental Association of Northern New Jersey . . . . . . 302

EDITORIAL.

Is Dentistry a Financial Success? . . . . . . 306

OBITUARY.

Resolutions of Respect to Dr. Charles A. Kingsbury . . . . . . 309

Dr. John Allen . . . . . . 309

BIBLIOGRAPHY . . . . . . 311

CURRENT NEWS.

Dental Society of the State of New York . . . . . . 314

Central Dental Association of Northern New Jersey . . . . . . 315

Harvard Odontological Society . . . . . . 315

College Commencements . . . . . . 317

Dental Patents . . . . . . 317

SELECTIONS.

A Case of Cocaine-Poisoning . . . . . . 318

Local Anæsthesia by Aquapuncture . . . . . . 320

Disinfectol . . . . . . 320

Therapeutics and Toxicology . . . . . . 320

